# Combinational approach of retrospective clinical evidence and transcriptomics highlight AMH superiority to FSH, as successful ICSI outcome predictor

**DOI:** 10.1007/s10815-020-01802-w

**Published:** 2020-05-20

**Authors:** Stavroula Lila Kastora, Olga Triantafyllidou, Georgios Kolovos, Athanasios Kastoras, Georgios Sigalos, Nikos Vlahos

**Affiliations:** 1 Department of Medical Sciences, University of Aberdeen, Aberdeen, UK; 2 Reproductive Medicine Unit, “Leto” Maternity Hospital, Mouson str. 7-13, 11524 Athens, Greece; 3 2nd Department of Obstetrics and Gynecology, Aretaieion Hospital, University of Athens, Vas. Sofias str. 7, 11528 Athens,, Greece; 4 Assisted Conception Unit “IAKENTRO”, Fragokklisias Str, 15125 Athens, Greece

**Keywords:** Assisted reproduction, Infertility, Transcriptomics, Retrospective clinical analysis, Bioinformatics

## Abstract

**Objective:**

Combination of transcriptomic and retrospective clinical data, to assess anti-Mullerian hormone (AMH) functionality at a cumulus cell level and evaluate AMH potential as a suitable marker for IVF outcomes (oocytes retrieved, number of day 3 embryos, gestation outcomes).

**Design:**

Raw RNA-sequencing data of cumulus cells sourced from younger (*n* = 10) patient group (group A) (age 29 (1 year of age), baseline FSH 7.4 (0.5 mIU/ml), AMH 4.67 (1.56 ng/ml)) and older (*n* = 10) patient group (group B) (age 43 (± 0.55 years of age), baseline FSH 8 (0.8 mIU/ml), AMH 1.07 (0.44 ng/ml)) were employed to derive transcriptomic differences among high vs. low AMH groups. We collected retrospectively patient data from 80 infertile patients selected according to pre-specified inclusion criteria.

**Setting:**

Publicly available raw RNA-sequencing data were retrieved from the SRA database of NCBI resource GEO Accession (GSM21575/35-44; GEO Accession: GSM21575/45-55). Retrospective data were collected from referrals to the Institute of Reproductive Medicine, Lito Hospital of Athens and the Institute of Life, Iaso Hospital of Athens, between the periods of March 2015 and April 2018.

**Intervention(s):**

A fixed human menopausal gonadotropin (hMG) antagonist protocol was used for all patients. All patients had serum AMH levels measured within a 3-month period prior to stimulation and serum levels of FSH and estradiol (day 2 of menstrual cycle; E2) (Clinical Trial code NV24042014).

**Main outcome measure(s):**

The primary outcomes were identification of transcriptomic variations among high (group A) vs. low (group B) AMH patients. Retrospective data primary outcomes were number of oocytes retrieved, fertilized successfully (grades A and B, day 2 embryos), and total number of day 3 embryos. Secondary outcome was live birth rate. Finally, we compared primary outcomes with AMH and FSH level as well as their genetic pathways (interacting genes) to demonstrate the predictive accuracy.

**Results:**

Essential players of the AMH signaling cascade, namely, *SMAD1*, *SMAD4*, *SMAD5*, *ALK1*, and L*EF1*, were significantly upregulated in group A (*n* 10) transcriptome. This biological clue was further supported by retrospective clinical data (*n* 80 participants), where AMH was positively correlated with both oocytes retrieved and fertilized as well as number of day 3 (grades A and B) embryos from patients undergoing IVF, in a statistically significant manner. AMH was further positive trend of association with successful pregnancy outcomes.

**Conclusion:**

Overall, this study offers new insight on AMH effects upon cumulus cells and new aspects on how AMH might promote oocyte integrity and embryo viability at a biochemical level as well as add to the current body of evidence supporting AMH clinical potential as a more sensitive marker of *IVF* outcomes in comparison with FSH, regarding numbers of oocytes received and high-quality day 2 and day 3 embryos.

**Electronic supplementary material:**

The online version of this article (10.1007/s10815-020-01802-w) contains supplementary material, which is available to authorized users.

## Introduction

Infertility presents a complex disorder with a vast range of implications spanning from medical and psychological to socioeconomic consequences, affecting 20–80 million people across the world. While advancing age, health status, and lifestyle factors present considerable causes of female infertility, unexplained infertility is estimated to affect approximately 15% of couples [1]. A multitude of research has been dedicated to identifying the most reliable ovarian reserve markers to predict the outcome of ovarian stimulation (OS), as it represents a critical cornerstone of assisted reproductive technologies (ART).]

**Fig. 1:**
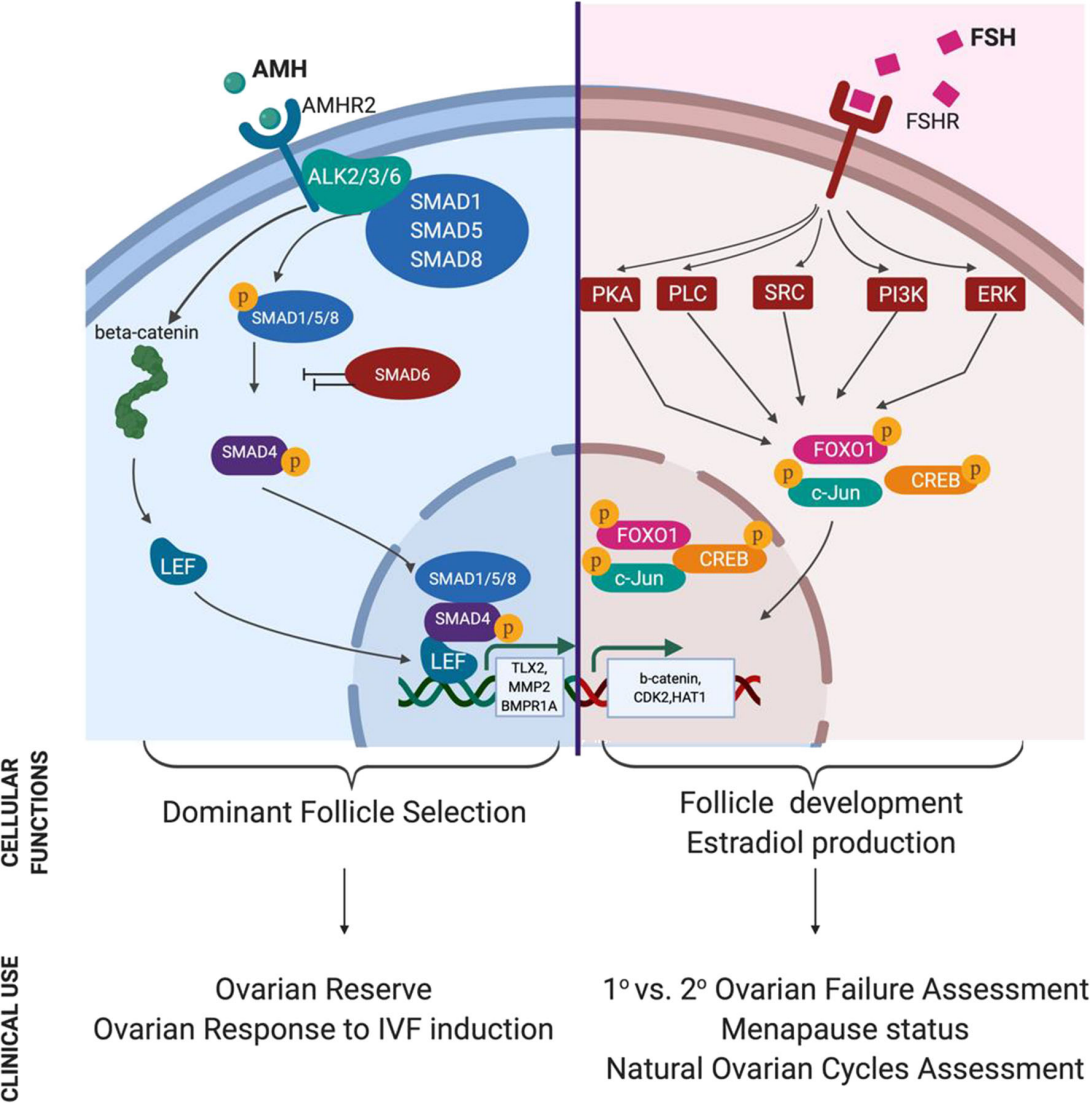
AMH and FSH signaling pathways, cellular outcomes, and main functions in IVF practice. (Blue cell) AMH binds to the Amhr2 receptor, leading to the formation of the Amhr2-Alk2, 3, and 6 type 1 transmembrane serine/threonine kinase complex. Activated complex initiates the phosphorylation of Smad proteins 1, 5, and 8. The latter bind to Smad4 allowing entry into the nucleus leading to follicle selection regulating genes. Smad6 which acts as a negative regulation of both BMP and TGF-beta/activin-signaling inhibits nuclear translocation of the activated complex leading to transcriptional suppression of Smad 1, 5, 8 targets. An accessory pathway through AMH stimulated b-catenin activation and subsequent binding to LEF-1 (Lymphoid Enhancer Binding Factor 1) further induces expression of Smad-dependent genes [3, 6, 7]. (Pink cell) FSH binding to the Fshr G protein-coupled receptor has been shown to activate the Gαs/cAMP/protein kinase A (PKA) signaling pathway [8]. Appl1 interacts with the FshR to trigger the PI3K signaling pathway while Src family members promote a similar activation in an FSH-dependant fashion, while triggering a similar response for the ERK cascade [9, 10]. Coupling of FshR to Gαh (tissue transglutaminase) leads to PLCδ activation [11]. Downstream effectors of these cascades, namely, Foxo1, Creb, and c-Jun, become activated through phosphorylation and translocate to the nucleus, leading to the expression of follicular development regulating genes as well as driving the expression of Estradiol. The FSH signaling cascades have been extensively reviewed in [12]

The new classification criteria of infertile women, according to their ovarian response to OS—the POSEIDON criteria—provide more precise guide in the management of these women than ESHRE (Bologna) criteria [2]. Although both existing criteria (Bologna and POSEIDON) use antral follicle count (AFC) and anti-Mullerian hormone (AMH) levels as the ovarian reserve tests, many clinicians still commonly employ FSH as marker of ovarian reserve in initial patient assessment prior to IVF cycle initiation [3]. AMH acts upon both granulosa cells and the oocyte to promote dominant follicle selection while FSH promotes follicle development and estradiol production [4, 5] (Fig. 1). Both hormones play central roles in the orchestration of gonadal function and consequently reproduction. While a multitude of studies have underlined the superiority of AMH as an ovarian reserve indicator [13–15], the effectiveness of either FSH or AMH as predictors of IVF outcomes and live birth rate remains obscure. Consequently, the identification of a non-invasive reliable predictor of OS success remains a significant goal in reproductive medicine.

At present, cumulus cell gene expression patterns and associated products present a promising target for the production of such a predictor. Folliculogenesis heavily relies upon the carefully coordinated cross-talk between oocyte associated cells, such as the cumulus and the oocyte. In the preovulatory stage (antral follicle), cumulus cells, a specialized type of granulosa cells, surround the oocyte [16]. Release of the antral follicle from the prophase I occurs after the LH surge, which precedes ovulation [17]. Cell cycle release and maturation of the preovulatory follicle require another process to occur, the “cumulus expansion,” during which production of hyaluronic acid and formation of a cumulus-oocyte binding extracellular matrix takes place [3]. Key mediators of this process are thought to be a multitude of signaling molecules spanning from extracellular signaling proteins such as GDF-9 (Growth Differentiation Factor 9), connexins, and PTX3 (Pentraxin 3), to transcription factors (FIGα) and catalytic mediators such as PDE3A (phosphodiesterase 3A) and HAS2 (hyaluronic acid synthase 2) [18–21]. This process partially triggers the oocyte maturation and the formation of a mature cumulus-oocyte complex, which results in an oocyte arrested at the metaphase of the second meiotic division, ready for ovulation and fertilization [3]. Accumulating evidence from a plethora of studies, although conducted upon model organisms, suggests that the orchestration of interaction of the above mediators present key events in successful folliculogenesis.

To assess the AMH effect upon the intricate interaction between cumulus cells and oocyte, we analyzed through transcriptomic approaches the differential expression of cumulus cell genes in younger patient (higher AMH levels, group A) vs. older (lower AMH levels, group B) groups with documented variations in AMH and FSH levels. Transcriptomic data were relayed upon the known FSH and AMH interactomes to underpin the effects of each hormonal driver upon this interaction. The AMH interactome was found to be significantly enriched in our differentially expressed genes with hits such as SMAD family members 1, 4, and 5 (*SMAD1*, *SMAD4*, *SMAD5*), *ALK1* (Activin A receptor like type 1), and *LEF1* (Lymphoid Enhancer Binding Factor 1), which are significant members of the AMH signaling cascade [4, 6, 7]. We further pursued this biological link in retrospective, clinical data of 80 patients, to investigate whether AMH presented a more sensitive indicator of IVF outcomes such as oocytes retrieved per cycle, oocytes fertilized (day 2, grades A and B embryos), and number of day 3 embryos. Secondary outcomes included positive correlation yet not statistically significant, of higher AMH levels with live births. This is the first analysis to combine biological and clinical data, with a bench-to-bedside approach, in support of AMH superiority in comparison with FSH as a positive predictor of positive IVF outcomes in terms of number of oocytes retrieved, grades A and B, day 2 and day 3 embryos.

## Materials and methods

### Patient data collection and demographics

#### RNA-sequencing

**Fig. 2:**
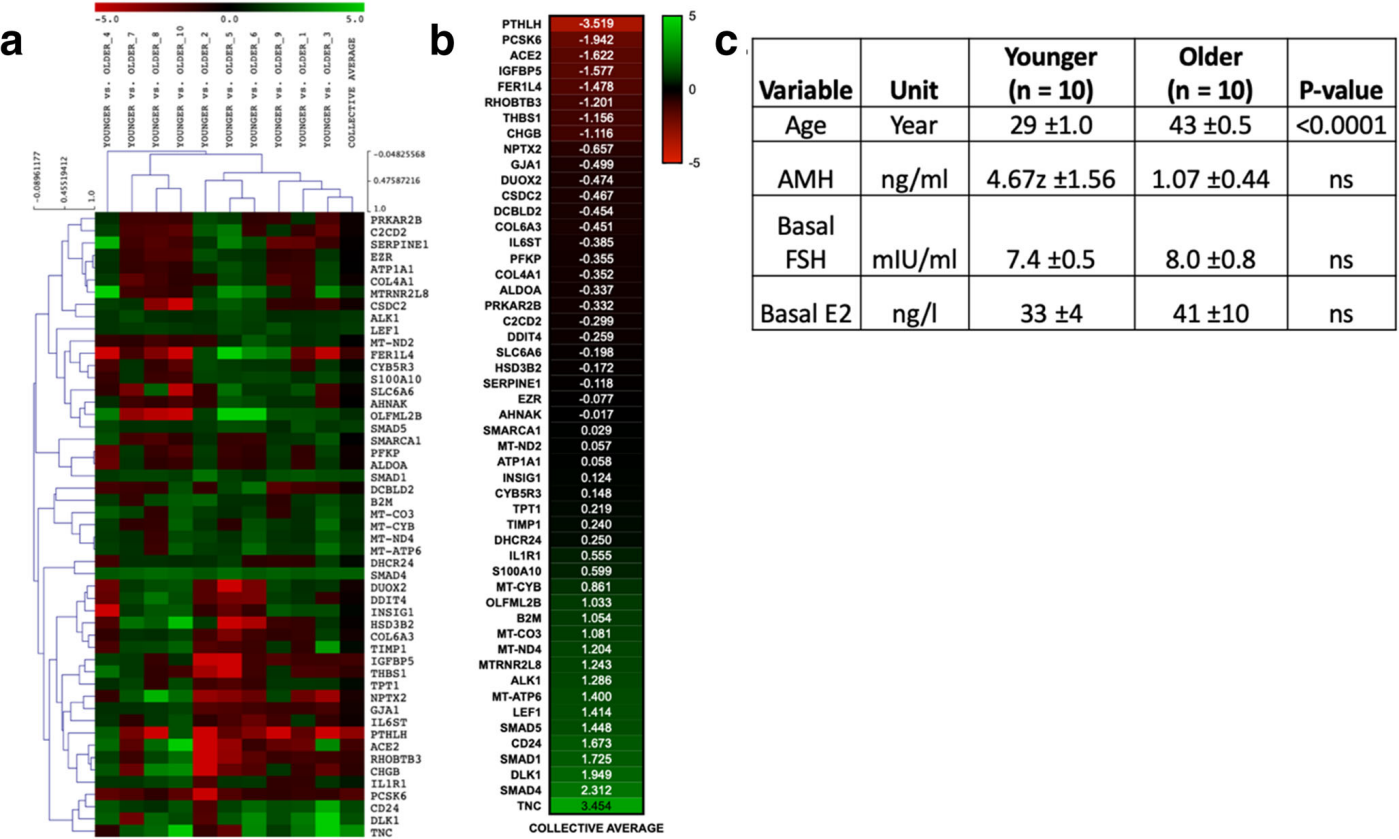
Differential expression of genes among randomly matched younger (*n* = 10) vs older (*n* = 10) patients. **a** Genes displaying over 1 degree of differential expression with statistical significance of < 0.05 were included in the feature list (Sup. Fig. 1). Unfiltered set of differential gene expression is provided in Sup. Table 3. Feature list genes were hierarchically clustered and normalized via average linkage, Euclidean point distance metric as defaults (Sup. Table 4). **b** Average expression among all samples for feature list is summarized. each heat map cell contains the average differential expression among all patient comparison samples. **c** Chart depicting sample demographics for significant values (standard error of the mean) such as AMH, FSH, E2, and age of patients [22]

Raw RNA-sequencing data of cumulus cells were sourced from younger (*n* = 10) patient group (group A) (age 29 (± 1 year of age), baseline FSH 7.4 (± 0.5 mIU/ml), AMH 4.67 (± 1.56 ng/ml)) and older (*n* = 10) patient group (group B) (age 43 (± 0.5 years of age), baseline FSH 8 (± 0.8 mIU/ml), AMH 1.07 (± 0.44 ng/ml)) [22] (Fig. 2c).

#### Retrospective clinical analysis

We collected, retrospectively, patient data from 80 infertile patients (with adequate and POR: poor ovarian reserve) selected according to the inclusion criteria stated below. All these women were referred to the Institute of Reproductive Medicine, Lito Hospital of Athens and the Institute of Life, Iaso Hospital of Athens, between March 2015 and April 2018. Inclusion criteria were as follows: (1) age (28–48 years of age), (2) two normal semen analyses, according to the 2010 World Health Organization criteria to exclude for male infertility [23], and (3) normal uterine cavity as well as tubal patency, which had been established via transvaginal ultrasound and hysterosalpingography or laparoscopy [24]. Ovarian reserve in all participants was assessed according to the guidelines of the American College of Obstetricians and Gynecologists [1]. Anonymized patient data can be found in Sup. Table 1. The eighty patients that met the inclusion requirements underwent 196 cycles (in total for all patients) of ovarian stimulation under the human menopausal gonadotropin (hMG, hMG-group) antagonist protocol. Day 2 grades A and B embryos (oocytes fertilized) and day 3 (grades A and B) embryos were assessed according to the ASEBIR embryo assessment criteria [25] according to morphology (number of cells, fragmentation %, symmetry, multinucleation, vacuoles, zona pellucida integrity). Embryos did not undergo pre-implantation genetic screening. The study was approved by the ethics committee of the Lito Maternity Hospital of Athens and the Institute of Life, Iaso Hospital of Athens. The study was also reported at the clinical trials registry (clinicalTrials.gov) with registration number NV24042014.

### Treatment schedule

All patients had serum AMH levels measured within a 3-month period prior to stimulation and serum levels of FSH and E2. AMH levels were assessed using the ultra-sensitive AMH/MIS ELISA kit (Ansh Labs), the automated access AMH assay (Beckman-Coulter), or the Elecsys® AMH Immunoassay (Roche). Thyroid function of the participants as well as serum prolactin was also conducted to exclude endocrine causes of infertility although not recorded in our dataset for analysis. All women were evaluated on the 2nd day of their menstrual cycle when a transvaginal ultrasound examination was performed by the same IVF specialist; serum levels of FSH and estradiol were measured concurrently. Providing that serum FSH was less than 16 mIU/ml and estradiol was less than 70 pg/ml, ovarian stimulation was initiated on day 3 with starting dose 150–300 IU of human menopausal gonadotropins (hMG) (MENOPUR®; Ferring Pharmaceutical Hellas AE) dependent on the age of the woman, BMI, baseline serum FSH and E2 levels, and serum AMH levels. All patients were re-evaluated on day 5 of stimulation (day 8 of the cycle), when a transvaginal sonogram was performed to evaluate follicular growth and serum estradiol levels were measured again. On day 6 of stimulation or when a leading follicle reached diameter of 14 mm, a commercially available GnRH antagonist, Cetrorelix acetate 0.25 mg/day, was initiated (Cetrotide; Merck Serono Hellas AE). Patients had serial evaluations as needed. When at least two follicles reached an average diameter of 18–20 mm, final oocyte maturation and ovulation was triggered by administration of a single dose of recombinant hCG (Ovitrelle®). Transvaginal ultrasound–guided follicular punctures and oocyte retrieval were performed 36 h post-hCG administration. The standardized ICSI protocol for oocyte manipulation and fertilization optimized from the Fertility Lito Unit (ISO: 9001:2008) was applied to all oocytes available for fertilization and one or a maximum of two embryos were transferred on day 3 after oocyte retrieval. All embryo transfers were performed by the same specialist under transabdominal ultrasound guidance to assess the endometrial cavity. Embryos were evaluated by the scoring system established by the Istanbul consensus workshop on embryo assessment [25]. Micronized progesterone (Utrogestan; Angelini Pharma ABEE) was used for supplementation of the luteal phase (200 mg four times daily vaginally) [26]. Serum beta-hCG was measured 12 days after the OPU (Ovum Pick Up) and clinical pregnancy was confirmed by ultrasound visualization of foetal heart rate 2 weeks later.

### Outcome measures

The primary outcomes measured were number of oocytes retrieved, oocytes fertilized [25], and total day 3 number of embryos. Secondary outcome was live birth rate. Finally, we compared primary outcomes with AMH and FSH level as well as their genetic pathways (interacting genes) to demonstrate the predictive accuracy.

#### Sample size calculation

Type I error rate was set at 0.05, type II error rate (*β*) = 0.2 as generally accepted standard for clinical research. Expected correlation coefficient was set at 0.4 for positive and − 0.4 for negative correlation. The minimum sample size was calculated as to achieve a *p* value of < 0.05 and include the expected correlation coefficient [27]. In this study, we recruited 80 patients that met our inclusion criteria, leading to 196 data entries (data entry was considered as a unit).

#### RNA-sequencing analysis

RNA samples were retrieved and sequenced using the Illumina HiSeq 2000 sequencing platform, as described in the SRA database of NCBI resource with study code (GEO Accession: GSM21575/35-44; GEO Accession: GSM21575/45-55) [22]. Patient allocation into age groups was conducted as described in [22], where difference observed among the two groups, e.g., group A (younger) 29 (± 1.0 years) vs group B (older) 43 (± 0.5 years), *p* value < 0.0001, was statistically significant, considering the limitations in the age groups that can be considered for ART. Covariate adaptive randomization [28] due to the small sample size with online randomization (http://www.graphpad.com/quickcalcs/index.cfm) of the patients assigned in each age group was employed to generate the 20 participants that agreed to provide genetic samples for RNA-sequencing in order to minimize selection bias. Cumulus RNA was extracted as described in [22]. Publicly available raw files (FASTA format) were aligned to *Homo sapiens* UCSC hg19 reference genome, trimmed for quality control (QC) analysis, and normalized (Sup. Fig. B) to provide meaningful data. Output data employed and processed in this study was analyzed successively in the following order through FLASH (V. 1.2.11.3), FastQC (v. 10.1), Trimgalore (v. 3.1), Samtools (v. 1.19), STAR aligner (v. 2.4), and Htseq (v. 5.4) on the Galaxy Community Platform [29] (usegalaxy.org) was conducted against the GCA_000001405.28 GRCh38.p13 *Homo sapiens* chromosome file provided by the NCBI Genome Database 21. Raw counts are provided in Sup. Table 2. Differential gene expression analysis was performed using Partek® Genomics Suite® software, version 6.6 Copyright ©; 2019. Gene counts (Sup. Table 2) were quantified and processed through GSA analysis to provide acceptable differential gene expression data (ratio, fold change, *p* value of group A vs. group B comparisons) for 15.601 genes (Sup. Table 3). Subsequent filtering of dataset for a minimum of 1-fold of change in expression and a *p* value of < 0.05 generated a feature list of 46 differentially expressed genes among all matched comparisons and processed with hierarchical Pearson clustering (Sup. Table 3, Fig. 2a). Sample normal distribution and pipeline of Partek analysis are shown in Sup. Fig. 1. GO_term analysis was performed in parallel through the STRING V.11 open platform 2019 [30] and the Cytoscape v. 2.5.4 Clue GO plugin [31]. Network construction was performed with Cytoscape V.3.7.2 freeware [32], Venn diagrams constructed using Venny (v. 2.1.0) online freeware [33], and heatmaps with TM4 MultiExperiment Viewer (MeV) (v. 4.9).

#### Statistical analysis

Statistical analysis was performed using GraphPad Prism (v. 8). A commercially available statistical program was used for the statistical analysis. A *p* value < 0.05 was considered statistically significant. Pearson correlation analysis was performed considering all variables were continuous (age, FSH/AMH/E2) measurements. All statistical analysis values (best-fit, slope calculation, 95% CI, Pearson Correlation *r* values/*p* values) are available on Figs. 5 and 7b.

## Results

RNA-sequencing data analyzed in this work enabled us to obtain a holistic view of the cellular changes regarding gene expression, in populations with contrasting AMH and FSH levels. As data were generated from patients and not obtained from a controlled, in vitro environment, variation among randomly matched comparisons (group A vs. group B) was expected. These variations may have been due to parameters that may have been recorded such as AMH, FSH, and E2 levels [Fig. 2a–c] or not (patient BMI/medical history, cell culture conditions, heat stress, etc.) affecting cumulus gene expression [34–36]. Thus, averaging among all groups was employed to allow for comparison among expressed genes (Fig. 2b).

**Fig. 3:**
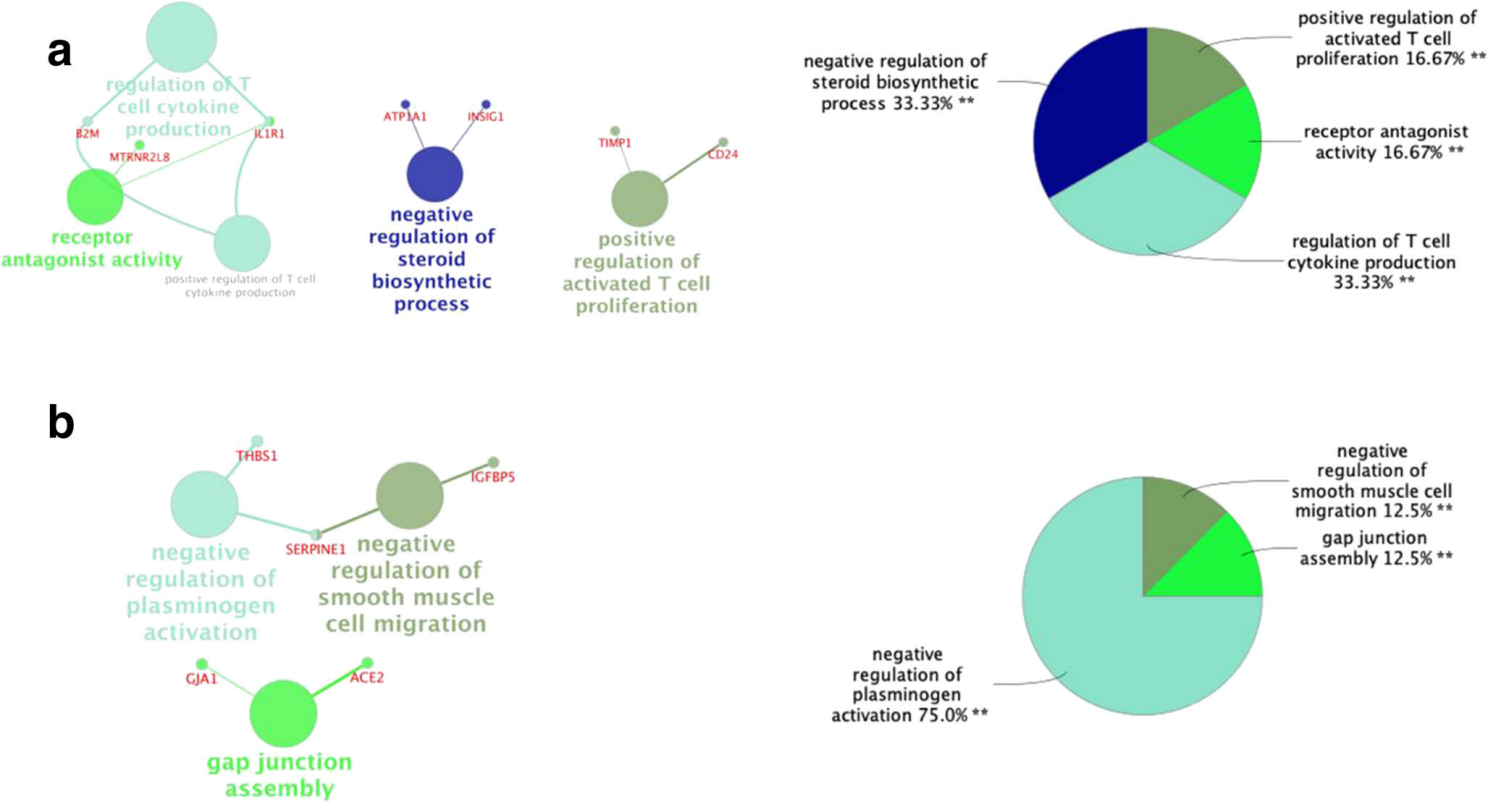
Biological analysis with Clue Go Cytoscape plugin of statistically significant, differentially expressed genes. **a** Upregulated genes with associated biological functions as identified through mean and normalization (Fig. 2b) of each younger vs. older patient comparison (*n* = 10) in a network and pie chart representation. **b** Downregulated genes with associated biological functions as identified through mean and normalization of each younger vs. older patient comparison (*n* = 10) in a network and pie chart representation. Genes that enrich each category with statistical significance *p* value < 0.05 appear connected to each GO_Biological Process node. Thickness of each edge represents statistical significance. Image was generated and adapted with Cytoscape plugin Clue GO V.2.5.4 [31]

We further explored the functionality of genes that displayed strong differential expression (up- or downregulation) with a bioinformatic approach (Fig. 3). Genes such as *PTHLH* were downregulated in the majority of patient comparisons, with a strongly negative average in the clustering comparison, while the opposite was true for genes such as *TNC* (Fig. 2b). Interestingly, the upregulated cluster of genes significantly enriched for T cell regulation of cytokine production (*IL1R1*, *B2M*, *MTRNR2L8*) as well as positive regulation of T cell proliferation (*TIMP1*, *CD24*), in support of previous literature underlining the immunological role of cumulus cells in protection of the associated oocyte and maintenance of pregnancy [37, 38]. Of note is that while the aforementioned T cell regulation has been associated with oocyte quality, protection, and pregnancy positive outcomes, the particular genes highlighted in this study have not previously been identified under this association. Further analysis highlighted upregulated genes (*INSIG1*, *ATP1A1*) that are associated with the negative regulation of steroid biosynthetic process, mediating regulation of cholesterol and sterol biosynthesis, thus averting increased steroidogenesis during early stages of follicular development [39]. On our downregulated gene cluster, statistically significant, biological processes, such as negative regulation of plasminogen activation (*SERPINE1*, *THBS1*), negative regulation of smooth muscle migration (*IGFBP5*), and positive regulation of gap junction assembly (*GJA1*, *ACE2*), were enriched. In murine models, active gap junction assembly as well as decreased smooth muscle migration biological processes have been shown to lead to ovarian failure as well through successful follicular development blockage [40]. Nonetheless, such association has not yet been made in human cell line models. Yet the present work, highlighting changes in these genetic pathways, should prompt future in vitro experiments in the field of oocyte development and maturation.

**Fig. 4:**
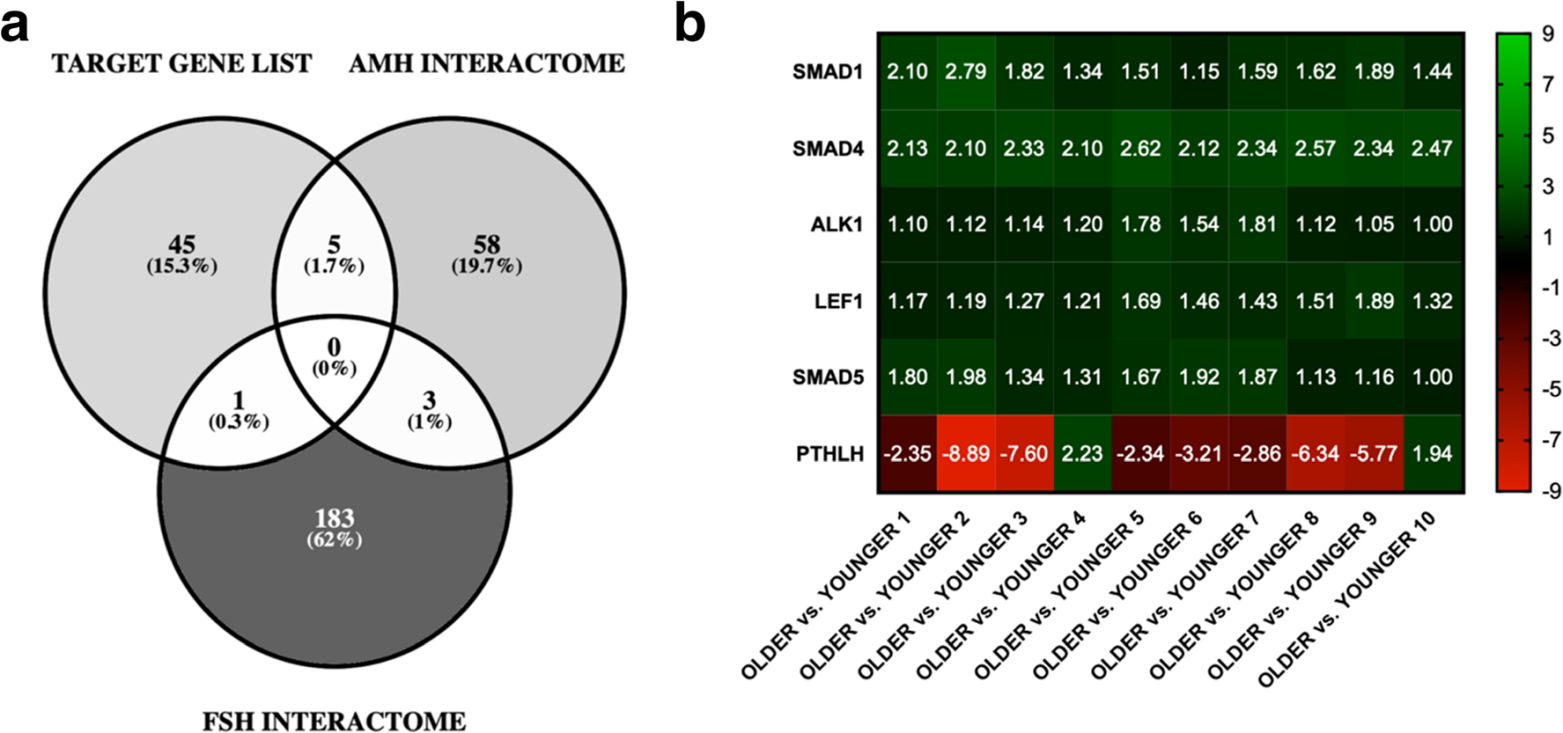
FSH and AMH interactome within feature, differentially expressed gene list. **a** Venn diagram (Venny 2.1.0) highlighting commonalities between AMH regulated genes vs. FSH regulated genes. Gene Card Database (https://www.genecards.org) was employed to identify genetic targets of both FSH (Hit word: Follicle Stimulating Hormone Subunit Beta. FSHB) and AMH. Target gene lists for each hormone where compared with the RNA-seq feature (target) list to identify potential areas of overlap. **a** One common element was identified between the “target gene list” and the “FSH interactome”: *PTHLH*. Five common elements (representing 1.7% of the target gene list) were identified between the “target gene list” and the “AMH interactome” which are as follows: *SMAD1*, *SMAD4*, *SMAD5*, *ALK1*, L*EF1.* Lastly, three common elements were identified between the “AMH interactome” and the “FSH interactome”: *ACVR2A*, *BMPR1B*, *SKIL.***b** Focussed heat map analysis of Venn diagram sourced, overlapping genes

Pursuing biological hints from the analyzed RNA-sequencing dataset, we wondered whether known hormonal markers (such as FSH and AMH) commonly used in clinical practice to assess ovarian reserve and follicular development affected gene expression in the examined cumulus cell samples. We sourced the FSH and AMH interactome (downstream gene targets, associated signaling pathways and interacting proteins) through the GeneCards, Human Gene Database [41], and cross-compared hits with our feature gene list (Fig. 4a). A 1.7% (*SMAD1*, *SMAD4*, *SMAD5*, *ALK1*, *LEF1*) of our target gene list was shared with the AMH interactome, in contrast to a 0.3% (*PTHLH*) shared with the FSH interactome. All AMH associated genes were upregulated, indicating these were expressed in higher levels in younger patients in comparison to older patients (Fig. 4b). In contrast, FSH-induced gene, Parathyroid Hormone Like Hormone (*PTHLH)*, was significantly (*p* value < 0.0001) lower in group A compared with group B suggesting that higher FSH levels, as often observed in older patients, may result in *PTHLH* downregulation, potentially underlining the role of calcium metabolism in anovulatory infertility [42].

**Fig. 5:**
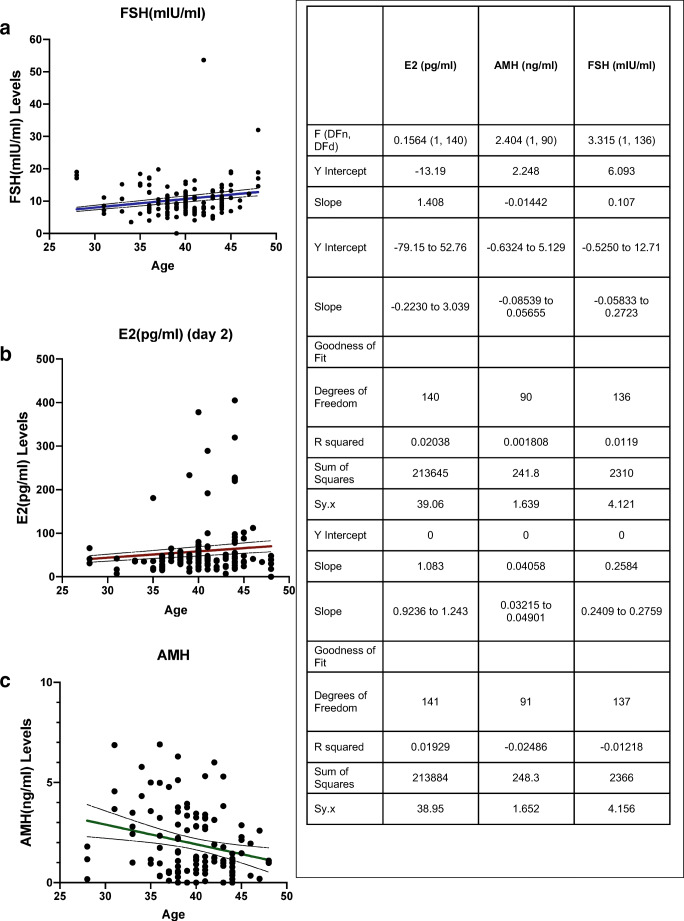
Linear regression lines of E2(pg/ml) (day 2) **a**, AMH (ng/ml) **b**, and FSH (mIU/ml) in relation to age of patient **c.** In the entirety of our data entries (*n* 196 entries; patients 80), FSH (mlU/mL), AMH (ng/ml), and E2 (pg/ml) day 2 were measured and analyzed with 95% confidence bands intervals (dotted curves) and non-linear fit lines as shown with colored edges in each graph. Tabular results of linear regression analysis shown in **a**–**c**

To assess AMH as an indicator of oocyte integrity and embryonal development potential, we collected retrospectively patient data (*n* = 80) as described in the “Materials and methods” section. Each IVF trial was noted as a separate event thus leading to 196 entries originating from 80 patients. The average number of IVF cycles was calculated at 2.45 (± 1.99 cycles). On stimulation day 2, E2 (35 ± 71.09 ng/l), FSH (7.91 ± 38.68 mIU/ml), and AMH (1.149 ± 1.26 ng/ml) levels were measured. AMH (Fig. 5c) displayed a decrease in concentration in the aging patient population while the opposite was noticeable for FSH (Fig. 5a), a finding in accordance with previous literature [43]. E2 levels displayed a visible increase in older patients [44] (Fig. 5b).

**Fig. 6:**
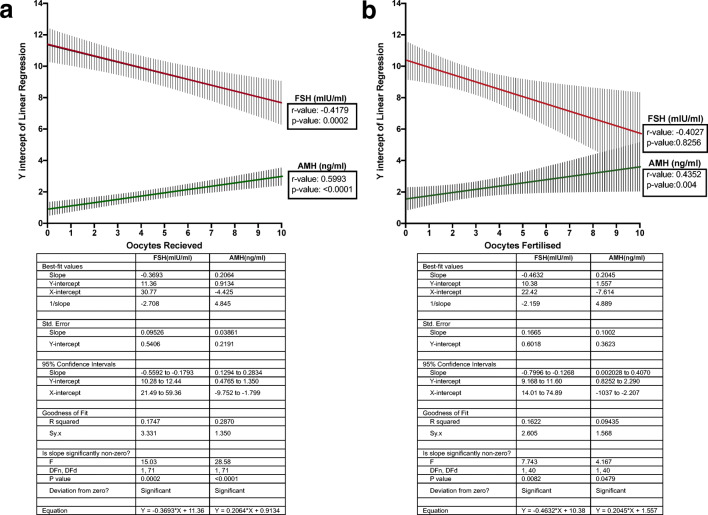
Simple linear regression and correlation analysis indicating that increased AMH displays statistically stronger positive correlation with the number of oocytes received and the final number of oocytes fertilized**.** Data of 80 individual patients were analyzed using Pearson correlation coefficients, computing *r* value (correlation coefficient) as well as *p* value for each correlation and linear regression slopes with 95% confidence bands of the resulting best-fit line. **a** Linear regression analysis and Pearson correlation coefficients and statistical significance (*p* value) for oocytes retrieved in correlation to FSH and AMH Y intercepts. **b** Linear regression analysis and Pearson correlation coefficients and statistical significance (*p* value) for oocytes fertilized, FSH and AMH Y intercepts

We then sought to investigate the correlation between FSH or AMH levels and oocyte count retrieved and number of embryos. Data from 73 patients were used, as 7 patients of our original cohort had opted out for embryo cryopreservation. Pearson correlation coefficients were calculated for both outcomes. In regard to oocytes retrieved, the *r* value of − 0.4179 for FSH indicated a negative, statistically significant (*p* value 0.0002) correlation while AMH, a positive, statistically significant (*p* value < 0.0001) correlation (*r* value 0.5993) (Fig. 6a). This result underlined that increased AMH held a stronger positive correlation with the increased number of oocytes received for patients undergoing assisted fertilization. Furthermore, increased AMH also displayed a stronger, in terms of significance (*p* value 0.004), positive correlation (*r* value 0.4352) with the number of oocytes successfully fertilized (Fig. 6b). On the other hand, increased levels of FSH displayed a negative correlation with the final number of day 3 embryos (*r* value − 0.4027), a result that was not deemed statistically significant (*p* value 0.8256). Furthermore, a similar pattern of correlation was observed between FSH (*r* value − 0.22; *p* value 0.02), AMH (*r* value 0.59; *p* value < 0.001), and the number of day 3 embryos obtained (Table 1). Overall, these results suggested that while increased FSH held a negative predictive value for the number of oocytes retrieved, in our patient group, AMH displayed a statistically stronger association with both oocyte retrieval, successful fertilization, and number day 3 embryos indicating a higher sensitivity as a marker of positive IVF outcomes.

**Table 1:** Tabular results of correlation matrix of associated variables with number of day 3 embryos retrieved

	Day 3 embryos vs. AGE	Day 3 embryos vs. FSH (mIU/ml)	Day 3 embryos vs. E2 (pg/ml) (day 2)	Day 3 embryos vs. AMH (ng/ml)	Day 3 embryos vs. oocytes received	Day 3 embryos vs. oocytes fertilized (day 2)
Pearson *r*	− 0.1764	− 0.2266	− 0.1548	0.5939	0.7965	0.8554
95% confidence interval	− 0.3315 to − 0.01199	− 0.3970 to − 0.04111	− 0.3293 to 0.02999	0.4546 to 0.7048	0.7272 to 0.8497	0.8040 to 0.8941
*R*-squared	0.03111	0.05135	0.02396	0.3527	0.6344	0.7317
*p* (two-tailed)	0.04	0.02	0.10	< 0.001	< 0.001	< 0.001
*p* value summary	*	*	ns	***	***	***
Significant? (alpha = 0.05)	Yes	Yes	No	Yes	Yes	Yes

Pearson correlation coefficients were calculated for each pair, with two-tailed *p* values to assess for statistical significance, Significance is categorized by the NJEM scale, e.g., 0.12 (ns), 0.033 *, 0.002 **, < 0.001 ***

**Fig. 7:**
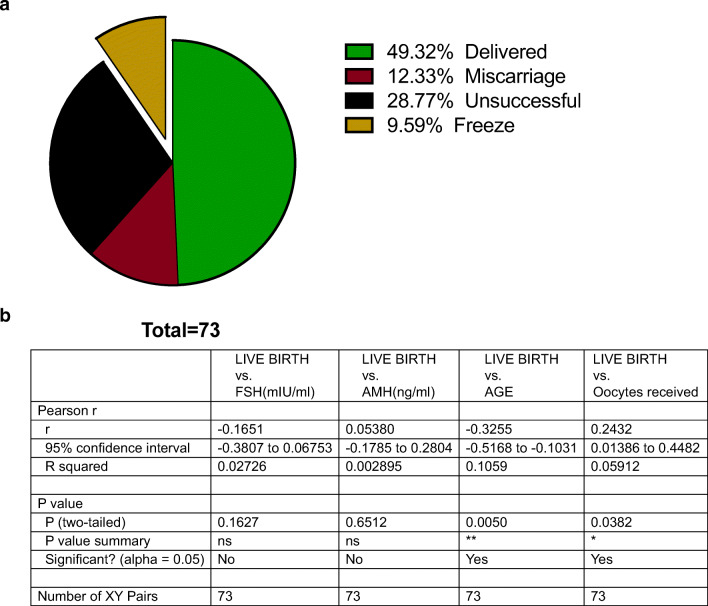
Outcomes of ovulation induction (*n* = 73) and correlation of variables with pregnancy outcomes. **a** Pie chart representing outcomes of ovulation induction including freeze cycles. **b** Tabular representation of Pearson correlation analysis of live birth (0 unsuccessful, 1 miscarriage, 2 embryo freeze, 3 live birth) with FSH, AMH, patient age, and oocytes received. Significance is categorized by the NJEM scale, e.g., 0.12 (ns), 0.033 *, 0.002 **, < 0.001 ***

Furthermore, we pursued to identify putative correlations between FSH and AMH induction levels and live birth rates (Fig. 7b). IVF was successful in 54.55% of the patients, excluding from the total population the women that opted out for embryo cryopreservation (freeze), 31.82% were unsuccessful (negative b-hCG test) and 13.64% miscarried (Fig. 7). Pregnancy outcomes significantly correlated (*r* value − 0.33, *p* value 0.003) with the age of the patient and the initial number of oocytes received (*r* value 0.24, *p* value 0.03) (Fig. 7b) and while AMH was positively correlated with live birth rate, the result was not deemed statistically significant in this study (*p* value 0.65).

## Discussion

In this work we sought to investigate the differences in younger (age 29) vs. older (age 43) women as stratified in previous work [22] and consequently differential gene expression in cumulus cells and assess putative associations with current clinical markers of oocyte integrity and thus successful IVF outcomes. Oocyte competence has been long associated with particular gene differential expression in cumulus cells. Nonetheless, the majority of these experiments were conducted on bovine samples, underlining the potential of delicate, species-specific differences [45, 46]. Pioneering studies in large patient samples (*n* 47) underlined the importance of particular genes (*STAR*, *COX2*, *AREG*, *GJA1*) expressed in human cumulus cells as putative predictors of oocyte quality and developmental potential (assessing the potential to reach a day 5/6 blastocyst level) [47]. Additionally, other studies have underlined the significance of pentraxin 3 expression as a putative marker of oocyte quality in human RNA-seq samples [48]. Nonetheless, grouping of patients according to age and cross-comparison with oocyte quality as outcome has not been previously attempted. As human studies do not allow for direct assessment of oocyte quality, genetic markers, cumulus cells provide a feasible alternative to infer oocyte developmental potential.

Here, we highlight the importance of gene expression such as *TNC* (Tenascin C), found to be significantly upregulated (3.45-fold difference) in our younger patient group (Fig. 2a, b). *TNC* has been implicated in guidance of migrating neurons, synaptic plasticity, and regeneration [49]. A previous study has also noted the increased expression of *TNC* in cumulus cells and localized the protein to extracellular matrix and membrane of murine cell lines [50]. While Tnc has not been deemed essential for the ovulation process in murine models as *tnc*−/− mice were found to be fertile, similar experiments have not been conducted in human samples [51]. On the other hand, calcium homeostasis has been shown to play a significant role in fertility while levels of PTH were noticeably increased in women with anovulatory infertility [42, 52]. These clinical observations were corroborated by our dataset as *PTHLH* expression was found to be significantly repressed in our younger group of patients (Fig. 2a, b). Interestingly, our biological markers expression matched inversely the increase of FSH levels and simultaneous decrease of AMH in our patient sample groups (Fig. 2c).

Bioinformatic approaches enabled us to elucidate the biological processes that were represented by our differentially expressed genes. The upregulation of genes, such as *TIMP1*, involved in the proliferation of T cells, as well as the *CD24* upregulation, modulator of B and T cell activation, underlined a novel approach by which cumulus cells might promote immune system modulation and consequent oocyte and embryo integrity (Fig. 3a). Furthermore, upregulation of *INSIG1* which modulates cholesterol metabolism and glucose homeostasis might either correspond to the necessity to decrease steroidogenesis during early stage, follicular development [39], or the previously noted impairment of *INSIG1* expression caused by ART manipulation techniques [53]. Intriguingly, genes (*SERPINE1*, *THBS1*, and *IGFBP5*), involved in inhibition of plasminogen activation and smooth muscle migration, were significantly downregulated in group A, indicating a relative higher expression in group B (Fig. 3b). This finding is supported by previous clinical studies that have concluded that higher tissue-plasminogen activator (t-PA) concentrations are observed in sub-fertile female patients [54]. Data from the present analysis underline putative biological agents that might be at interplay; nonetheless, the exact mechanism of this process remains to be elucidated and presents ground for further research.

We further pursued putative associations of the biological gene targets with increased concentrations of currently used clinical markers of fertility, FSH and AMH. As the AMH interactome was found to be more extensively overlapping with our feature gene list, we hypothesized that AMH might present a better marker of positive IVF outcomes in comparison to FSH. Our clinical data come in support of previous literature in terms of AMH superiority in predicting oocytes retrieved with a positive correlation [14, 55] (Fig. 6a). Nonetheless, our results could not statistically confirm a strong (*p* value < 0.05) association of AMH with live birth (Fig. 7b), as primarily AMH functions as a marker of reproductive longevity rather than predictor live birth. This result may well be due to patient numbers as AMH/live birth correlation was a secondary outcome of this work and thus patient sample was not calculated upon the statistical significance of this particular correlation. Furthermore, our data first provided evidence of AMH superiority in regard to number of grades A and B, day 3 embryos (Fig. 6b).

Of note was that an obvious limitation of this study was the unmatched patient RNA-seq, samples with the clinical data. A limitation was to be expected by the structure of this study due to its retrospective character. Nonetheless, matched RNA patient samples and extended registered values in terms of patient demographics (e.g., parameters such as TSH, free T4/T3, progesterone levels, prolactin, and cortisol [56, 57] could shed new light in the biological factors between cumulus cells and oocyte quality factors while decreasing discrepancies due to patient ethnic origin. Furthermore, AMH assays were conducted in patient selected, private diagnostic centers. Thus, the authors were only able to collect data of AMH measurements rather than suggest a specific AMH kits. Consequently, a standardized singular AMH kit selection and ideally increased numbers of patients could increase statistical power potentially enabling current, not-significant associations to emerge as significant, e.g., AMH levels and live births. Lastly, standardization of the kits used to measure hormonal levels could increase the power of this study by decreasing disparities due to kit sensitivity variations.

## Conclusions

Besides the obvious benefits in predicting oocyte quality and embryo developmental potential from AMH levels and cumulus cell markers in respect to ART efficacy, another point of clinical interest might be the decreased probability of high-order multiple gestations. The negative impact of multifetal gestation on both embryo and mother has been extensively documented [58]. As efficiency of ART approaches increase and double embryo transfer (DET) cycles become a trend due to clinical outcomes and cost-effectiveness, a drift towards higher order pregnancies has been observed [59]. Being able to predict embryo developmental potential and live birth probability would further decrease risks associated with such pregnancies, such as miscarriage, post-partum hemorrhage, and mortality, as well as neonatal prematurity, disability, and mortality. Clinical and biological findings of this study suggest that AMH and its interactome in cumulus cells might present such clinical predictive solutions without compromising oocyte integrity, a statement that remains to be fully elucidated by aforementioned experimental approaches. Overall, this study offers new insight on AMH effects upon cumulus cells and new aspects on how AMH might promote oocyte integrity and embryo viability at a biochemical level as well as underline AMH clinical potential as a more sensitive marker of *IVF* outcomes than FSH.

## Electronic supplementary material

ESM 1 (XLSX 28 kb)

ESM 2 (TXT 2039 kb)

ESM 3 (DOCX 96 kb)

ESM 4 (XLSX 6387 kb)

ESM 5 (TXT 5 kb)
