# Supplementary material for: Combinational approach of retrospective clinical evidence and transcriptomics highlight AMH superiority to FSH, as successful ICSI outcome predictor
Source: J Assist Reprod Genet. 2020 May 20;37(7):1623–35. doi: 10.1007/s10815-020-01802-w (PMC7376803; doi:10.1007/s10815-020-01802-w)
Supplement: Supplementary file 3 — (DOCX 96 kb) [file 10815_2020_1802_MOESM3_ESM.docx]

**Manuscript Title:** Combinational approach of retrospective clinical evidence and transcriptomics highlight AMH superiority to FSH, as successful ICSI outcome predictor.

**Supplementary Figures**


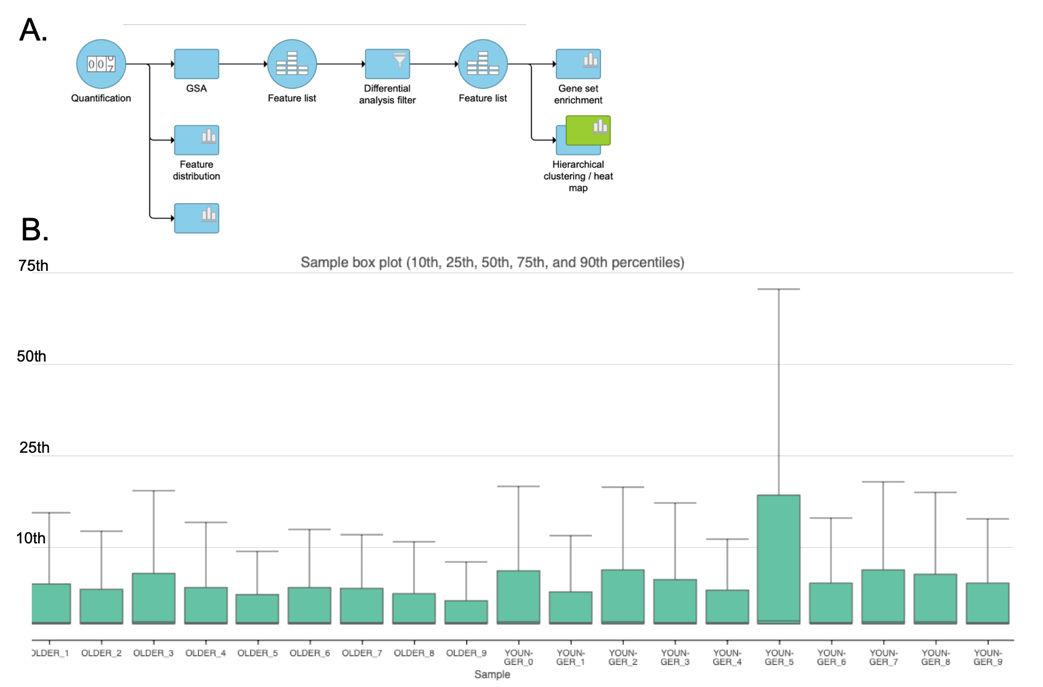


**Sup. Figure 1: Partek Flow Pipeline of RNA sequencing analysis and sample quantification results.** (A). Post alignment samples (n=20) (ERX1104364) were quantified for Feature distribution and processed to remove poor quality reads and for assigned to groups for Gene Set Analysis (GSA). Resulting data were assessed for quality and off target reads as shown in (B). Sample with greatest variation in reads was “Younger_5”, nonetheless sample was included in downstream analysis to decrease level of stratification.
